# Human T-Lymphotrophic Virus Type-I: A Unique Association with Myelopathy in Sjögren’s Syndrome

**DOI:** 10.4172/2327-5073.1000e123

**Published:** 2015-01-05

**Authors:** Alexandria Voigt, Cuong Q Nguyen

**Affiliations:** 1 Department of Infectious Diseases and Pathology, College of Veterinary Medicine, University of Florida, 2015 SW 16th Ave, Gainesville Florida, USA; 2 Center for Orphan Autoimmune Disorders, University of Florida College of Dentistry, 1600 SW Archer Rd, Gainesville, Florida, USA

## Editorial

Microbes play the most intricate and crucial role in the education of the immune system. Exposure to pathogens elicits specific immune response to clear the infection. After elimination of infectious pathogens, the immune cells undergo apoptosis during the contraction phase, and the remaining cells return to the quiescent memory phase [1]. During the primary and secondary responses, some of the immune cells fail to distinguish self and non-self. The failure to discriminate self and non-self entities triggers autoimmunity [2]. For decades, mounting evidence has supported the associations between infectious agents and autoimmune diseases. Two notable examples are rheumatic fever with *Streptococcus pyogenes* infection [3] and multiple sclerosis with Epstein-Barr virus (EBV) and Influenza type A virus [4]. The renewed interest in linkage of infectious disease and autoimmunity has thrived with primary autoimmune diseases associated with secondary peripheral neuropathy such as Sjögren’s syndrome (SjS). SjS is an autoimmune disease which is generally categorized by sicca symptoms, the presence of (anti-SSA or anti-SSB) autoantibodies, and/or lymphocytic infiltration into the salivary gland [5,6]. There are a number of viruses which have been shown to associate with primary SjS [7-10]. However, lymphotrophic virus-1 (HTLV-1), which is a retrovirus originally identified as an etiological agent in T cell leukemia [11], is hypothesized to be correlated with the manifestation of myelopathy in some SjS patients and whose treatment efficacy has been variable [12].

In cases of infection, onset of myelopathy has been acute with patients feeling neck pain followed by parathesia, numbness or pain in their extremities which in some cases is followed by paraplegia [13]. There is debate as to whether HTLV-1 infection can act as an environmental trigger to cause SjS, however it remains unclear. Patients may not actually know that they have SjS until they seek out medical attention for these symptoms and patients with SjS can develop myelopathy without HTLV-1 infection. Ironically, patients with SjS would not usually be tested for HTLV-1 unless myelopathy occurred and patients with HTLV-1 infection would not be tested for SjS unless myelopathy occurred; some patients with SjS test negative for anti-SSA or anti-SSB autoantibodies and may lead to a negative diagnosis when SjS is present.

In one study [11], of ten patients presenting with HTLV-1 associated myelopathy (HAM), six were diagnosed with SjS and two were suspected to have it. Since all of the HAM patients also showed lymphocytic infiltration in the salivary glands (whether anti-SSA or anti-SSB autoantibodies were present), it becomes difficult to diagnose SjS as that is one of the qualifying criteria. Either salivary gland infiltration is a symptom of HTLV-1 associated exclusively to HAM, or SjS patients exclusively exhibit HAM. Further examination and larger cohorts of patients would be necessary to confirm which of these two options is correct.

Use of animal models is another viable option for studying this relationship. In one transgenic rat model (HTLV-1 *env-pX*), the *env-pX* gene was inserted into the WKAH strain [14] and rats developed SjS-like symptoms along with an array of other symptoms characteristic of autoimmune diseases [15], but manifestation of myelopathy was not studied. Further examination of this line, focusing on myelopathy exclusively may yield an answer as to whether or not HAM is present only in SjS patients, but this may be difficult as they are prone to developing other autoimmune disorders. Complexing SjS with another autoimmune disorder may convolute the findings as they may either exacerbate or mask symptoms.

To further understand HAM moving forward, it will probably become necessary to correctly diagnose patients with or without SjS. For HAM in SjS patients combined treatment with a corticosteroid and anti-viral has shown improvement in the patients’ condition without resolving the lesion [16]; reversal of parathesia, numbness, pain or paraplegia may occur. Further examination of HAM both in a clinical setting and utilizing animal models may be necessary to fully understand if and why HAM presents exclusively in SjS patients.
